# Diffuse Recurrence of Hepatocellular Carcinoma After Liver Resection: Transarterial Chemoembolization (TACE) Combined With Sorafenib Versus TACE Monotherapy

**DOI:** 10.3389/fonc.2020.574668

**Published:** 2020-12-17

**Authors:** Wang Yao, Miao Xue, Mingjian Lu, Yu Wang, Yue Zhao, Yanqin Wu, Wenzhe Fan, Jiaping Li

**Affiliations:** ^1^ Department of Interventional Oncology, the First Affiliated Hospital of Sun Yat-Sen University, Guangzhou, China; ^2^ Department of Radiology, Affiliated Cancer Hospital & Institute of Guangzhou Medical University, Guangzhou, China

**Keywords:** transarterial chemoembolization, sorafenib, hepatocellular carcinoma, diffuse recurrence, liver resection

## Abstract

This study aims to compare the effectiveness and complications of transarterial chemoembolization (TACE) combined with sorafenib (S-TACE) and TACE monotherapy in HCC patients with diffuse recurrence (DR). This retrospective study was approved by our hospital ethics committee, and all patients provided informed consent. We retrospectively enrolled 356 DR patients from January 2005 to December 2014, who underwent either S-TACE or TACE monotherapy. Treatment complications, overall survival (OS) and progression-free survival (PFS) were evaluated. Survival curves were constructed using the Kaplan-Meier method and compared using a log-rank test. Our results found a significant difference between S-TACE and TACE monotherapy in the PFS and OS of HCC patients with early diffuse recurrence (EDR) (*p*=0.011 and 0.049, respectively). Patients with late diffuse recurrence (LDR) who underwent S-TACE had longer OS (median 24.0 vs. 16.0 months; *p*=0.044) compared with those in the TACE monotherapy group. Subgroup analysis revealed that S-TACE therapy resulted in higher OS of EDR patients with tumors > 5 cm and HBV-DNA >100 (*p*=0.036 and 0.035, respectively), compared with patients given TACE monotherapy. S-TACE therapy also resulted in better OS in LDR patients with AFP≥400 ng/ml, AFP<400 ng/ml, TB<28 g/L, TB>28 g/L, and a maximum tumor diameter < 5 cm (p=<0.001, 0.042, <0.001, <0.001, and <0.001, respectively). The rate of major complications in patients who underwent S-TACE was not significantly different to those who underwent TACE monotherapy (33.5% vs. 28.2%, *p*= 0.69). Overall, patients given S-TACE had better OS in both EDR and LDR patients, but only EDR patients had better PFS.

## Introduction

Hepatocellular carcinoma (HCC) is the fifth most common type of tumor and the third largest cause of cancer-related deaths (1). Liver resection is a curative treatment method for HCC, however, only 9%–27% of HCC patients are eligible for surgical resection (2). Although radical hepatectomy can be therapeutically effective for small HCC, the recurrence rate remains high (3). Diffuse recurrence (DR) is defined as 10 or more new recurrent nodules with ill-defined tumor margins (4, 5). DR is divided into early diffuse recurrence (EDR) and late diffuse recurrence (LDR) based on the time to recurrence (6). According to the 2018 European Association for the Study of the Liver (EASL) HCC guidelines, DR is classified by multinodular recurrence, with transarterial chemoembolization (TACE) recommended as the optimal treatment. However, the efficacy of TACE is limited, and quick recurrence following this treatment can result in a worse prognosis according to the study of Choi et al. (7). So, new therapies are urgently needed.

In most circumstances, chemoembolization is the optimal treatment for multinodular recurrent HCC (8). TACE can prolong the survival of patients by preserving the liver function and treating multinodular asymptomatic tumors without macroscopic vascular invasion or extrahepatic spread (9, 10). Sorafenib, an oral multikinase inhibitor with antiproliferative and antiangiogenic activities, is recommended for patients with advanced-stage HCC (11, 12). Chao et al. combined TACE with sorafenib (S-TACE) in patients with multinodular, unresectable HCC. Of their patients, 81.5% did not have vascular invasion or extrahepatic spread. Their results found that S-TACE was well tolerated and efficacious in patients with multinodular HCC without vascular invasion and extrahepatic spread (13). However, the effects of S-TACE in patients with DR remains unknown.

Few reports have focused on studying or developing a treatment strategy for DR in patients with HCC who have undergone previous liver resection. The aim of this retrospective study was to compare the effectiveness and safety of S-TACE and TACE monotherapy in HCC patients with DR who have undergone a previous liver resection.

## Materials and Methods

### Ethical Statement

Written informed consent was obtained from each patient prior to the treatment. The patients were sufficiently informed of the risks, benefits, and alternatives to both S-TACE and TACE monotherapy. The study protocol followed the ethical guidelines of the 1975 Declaration of Helsinki (as revised in Brazil in 2013). This retrospective study was approved by the institutional review board of our hospital.

### Patient Selection

**Figure 1:**
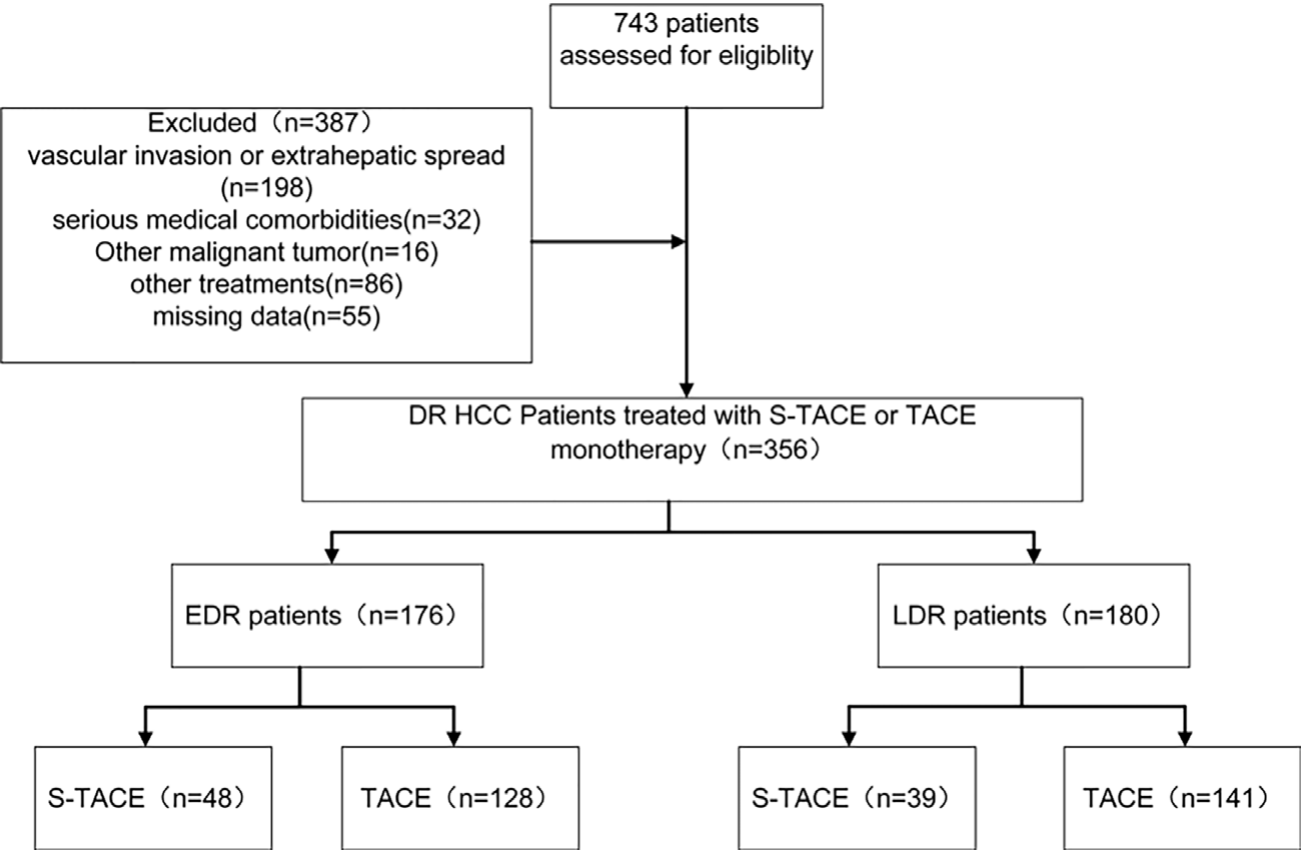
Flowchart showed patient selection.

HCC was diagnosed according to the European Society of Digestive Oncology (14) and classified based on the Barcelona-Clinic Liver Cancer (BCLC) staging classification (15). The inclusion criteria were as follows: (a) patients with their first recurrence after liver resection; (b) aged 18–75 years; (c) with 10 or more new recurrent nodules; (d) with Eastern Cooperative Oncology Group performance status of 0 or 1; (e) with Child-Pugh classification of A or B. The exclusion criteria was: (a) patients with extrahepatic spread; (b) with serious medical comorbidities, such as dysfunction of the heart or kidneys, severe coagulation disorders, etc.; (c) with other current malignancies or a history of other malignancies besides HCC; (d) with vascular invasion; (e) patients who had undergone other treatments before this study (**Figure 1**).

### Transarterial Chemoembolization Procedure

All TACE procedures were performed by 1 of 3 interventional physicians. A 5F catheter (Terumo, Tokyo, Japan) or a 2.7F microcatheter (Renegade Hi-Flo Straight, Boston scientific, Natick, Mass; Progreat, Terumo, Tokyo, Japan) was employed for tumor-feeding artery superselective therapy. An emulsion of 5–20 ml lipiodol (Lipiodol; Guerbet, Aulnay-Sous-Bois, France) and 20–60 mg epirubicin (Pharmorubicin; Pfizer, New York, USA) were administered into the tumor-feeding vessels. The specific dose of lipiodol was determined based on the tumor number and volume. Then, 350–560 mm absorbable gelatin sponge particles (Gelfoam; Hangzhou Pharmaceutical, Linan, China) were administered into the tumor-feeding vessels. The embolization finishes when the radiocontrast agent stopped flowing for 5 cardiac cycles.

### Sorafenib Management

Sorafenib therapy (daily dose, 400 mg BID) was initiated 2-5 days after the initial TACE and continued until the emergence of intolerance, refusal, and tumor progression. Sorafenib dose reduction was determined based on the presence of toxicity. If grade 3 or 4 adverse events (AEs)-defined by the National Cancer Institute Common Terminology Criteria for Adverse Events (16)-occurred, a dose adjustment (400 mg once daily) was performed until AEs were alleviated or eliminated. If grade 3 or 4 AEs continued after dose adjustment, sorafenib treatment would be halted until AEs were alleviated or eliminated.

### Survival, Tumor Progression, and Safety

In this study, the primary endpoint evaluated was overall survival (OS), defined as the time HCC recurrence was diagnosed to the date the patient died for any reason. The secondary endpoint was progression-free survival (PFS), defined as the time HCC recurrence was diagnosed to the date on which the tumor progressed. HCC progression was defined as the appearance of local tumor progression, new HCC nodule, vascular invasion, or extrahepatic spread according to contrast-enhanced dynamic CT or MR imaging results. Treatment responses were divided according to the mRECIST standard into CR, PR, SD, and PD (17). Contrast-enhanced dynamic CT or MR results were discussed and confirmed by two radiologists.

We assessed the safety and toxicity of TACE and oral sorafenib administration in all patients, using the National Cancer Institute Common Terminology Criteria for Adverse Events. Grade 3–4 AE was defined as an event leading to substantial morbidity and disability (resulting in the unexpected loss of an organ), which resulted in increase in the level of care, hospital admission, length of hospital stay, or led to the adjustment or discontinuation of treatment protocols. Grade 3–4 AEs were considered major complications, other complications were regarded as minor.

### Follow-Up

All patients were followed monthly for the first 3 months, then every 3 months until 2 years after TACE, and annually thereafter. Follow-up assessments included a detailed medical history, physical examination, laboratory tests, and chest and abdominal contrast-enhanced CT or MRI examination. When tumor progression occurred, the decision to perform repeated TACE was made by an MDT group.

### Statistical Analyses

All statistical analyses were performed using SPSS software (version 24.0; SPSS, Chicago, IL*, USA*). Continuity correction and independent sample *t-test*s were used to analyze the quantitative data including age, ALB, TB, and maximal tumor diameter. Pearson *x* ^2^ and Fisher’s exact tests were applied for qualitative data such as sex, cause of HCC, liver cirrhosis, AFP, HBV-DNA, ECOG, Child-Pugh class, and the incidence of complications. The cutoff value was calculated using R (TIBCO, Silicon Valley, CA). OS and PFS were calculated using the Kaplan-Meier method. The Kaplan-Meier survival estimates were compared using a *x* ^2^ statistic with a Log-Rank weighting scheme. Univariable and multivariable analyses were performed using the Cox proportional hazard regression model. All statistical tests were two-tailed, and a *p-value* <0.05 indicated a significant difference.

## Results

### Optimal Cutoff Value for Distinguishing Early and Late Diffuse Recurrence

**Figure 2:**
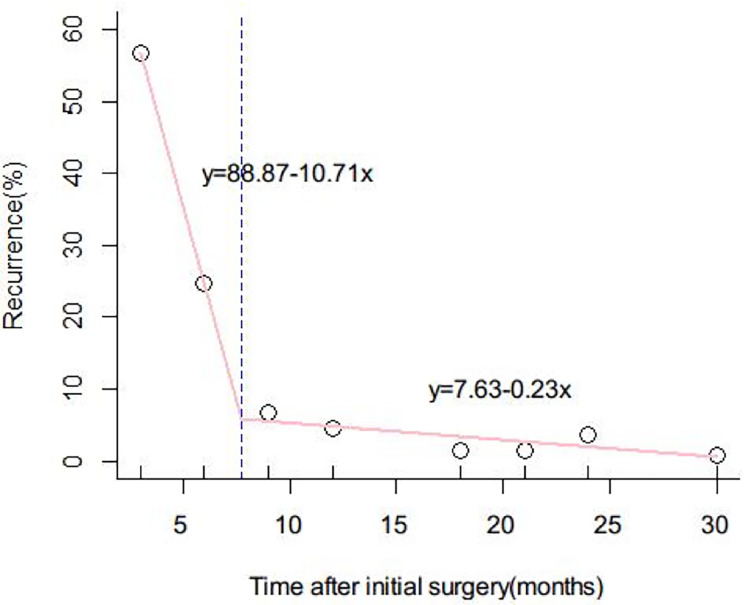
Determination of the optimal cutoff value for early and late diffuse recurrence of hepatocellular carcinoma (HCC). The function of the two lines was y = 88.87 – 10.71x and y = 7.63 − 0.23x, respectively. The intercept value of the two lines was 8 months. 8 months was therefore defined as the optimal cutoff value to differentiate early and late diffuse recurrence of HCC.

Recurrence was evaluated every 5 months to determine the optimal cutoff value for distinguishing between EDR and LDR. Eight months was found to be the optimal cutoff value, as shown in **Figure 2**. Clinicopathological data and outcomes after recurrence were analyzed and compared between the EDR and LDR groups.

### Patient Characteristics

Between January 2005 and December 2014, 356 patients at our hospital developed DR after initial liver resection. Follow-up data were collected until December 30, 2017. Patients were divided into 2 groups (the EDR group and LDR group) according to recurrence type. In the EDR group, 48 cases (27%) were in the S-TACE group and 128 cases (73%) were in the TACE monotherapy group. In the LDR group, 39 cases (22%) were in the S-TACE group and 141 cases (77%) were in the TACE monotherapy group. Median age of the EDR patients was 55.0± 11.7 and 56.0±11.9 years in S-TACE and TACE monotherapy groups, respectively. The median age of the LDR patients was 52.0±12.8, 57.0±12.3 years in S-TACE and TACE monotherapy groups, respectively. There was no significant difference in the baseline characteristics of the EDR and LDR groups (**Table 1**, **Table 2**). All baseline characteristics were collected before TACE. The median follow-up time was 52 months (range, 2–62 months) in the EDR group and 63 months (range, 3–86 months) in the LDR group. There were 123 cases (70%) in the EDR group and 96 cases (53%) in the LDR group where the patient died during the follow-up period. The mean duration of sorafenib treatment was 11 months (range, 1–23 months).

**Table 1:** Baseline patient characteristics of early diffuse recurrence (EDR) patients.

Characteristic	S-TACE (n=48)	TACE (n=128)	P value
Sex			0.789
Male Female	42(87.5) 6(12.5)	110(85.9) 18(14.1)	
Age(y)			0.565
Median±SD Range	55.0±11.7 26–77	56.0±11.9 22–78	
Cause of HCC			0.514
HBV HCV Alcohol Other	39(81.3) 4(8.3) 1(2.1) 4(8.3)	109(85.2) 9(7.1) 3(2.3) 7(5.4)	
Liver cirrhosis	33(68.8)	101(78.9)	0.160
ALB(g/L)			0.124
Median±SD Range	36.0±6.7 27-73	37.0±6.3 24-66	
TB (umol/L)			0.618
Median± SD Range	25.5±6.3 11–44	27.0±13.4 5–90	
AFP(ng/ml)			0.053
≥400 <400	21(43.8) 27(56.3)	78(60.9) 50(39.1)	
HBV-DNA ≥100 <100	31(64.6) 17(35.4)	53(41.4) 75(58.6)	0.051
ECOG			0.891
0 1	42(87.5) 6(12.5)	111(86.7) 17(13.3)	
Child-Pugh			0.508
A B	41(85.4) 7(14.6)	114(89.1) 14(10.9)	
Maximal tumor diameter(cm)			0.181
Median ± SD Range	5.0±2.6 2.0-13.0	4.0±2.9 1.0-13.0	

Except where indicated, data are numbers of patients. Data in parentheses are percentages and were calculated by using the total number of patients in each group as the denominator.

**Table 2:** Baseline patient characteristics of late diffuse recurrence (LDR) patients.

Characteristic·	S-TACE (n=39)	TACE (n=141)	P value
Sex			0.247
Male Female	36(92.3) 3(7.7)	127(90.1) 14(9.9)	
Age(y)			0.428
Mean±SD Range	52.0±12.8 22–76	57.0±12.3 22-78	
Cause of HCC			0.613
HBV HCV Alcohol Other	33(84.6) 3(7.7) 1(2.6) 2(5.1)	124(87.9) 6(4.3) 4(2.8) 7(5.0)	
Liver cirrhosis	31(79.5)	110(78.0)	0.844
ALB(g/L) Mean±SD Range	36.0±4.4 27–47	35.0±6.3 24–66	0.262
TB (umol/L)			0.088
Mean ± SD Range	21.0±10.1 7.9-39.0	14.9±9.8 7.6-41.0	
AFP(ng/ml)			0.833
≥400 <400	19(48.7) 20(51.3)	66(46.8) 75(53.2)	
HBV-DNA(U/ml)			0.062
≥100 <100	10(25.6) 29(74.4)	59(41.8) 82(58.2)	
ECOG			0.247
0 1	33(84.6) 6(15.4)	107(75.9) 34(24.1)	
Child-Pugh			0.276
A B	32(82.1) 7(17.9)	125(88.7) 16(11.3)	
Maximal tumor diameter(cm)			0.279
Mean ± SD Range	6.0±3.3 1.0–15.0	5.0±3.2 1.0–16.0	

Except where indicated, data are numbers of patients. Data in parentheses are percentages and were calculated by using the total number of patients in each group as the denominator.

### Recurrence-Free Survival and Overall Survival

**Figure 3:**
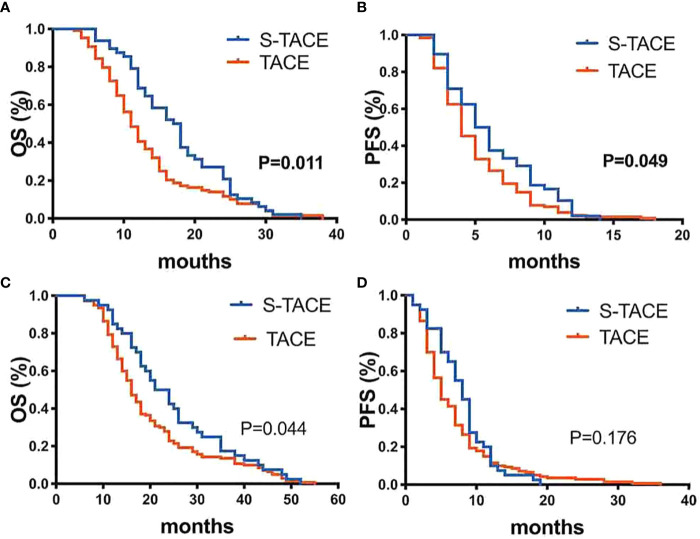
Kaplan-Meier curves showed overall survival **(A)** and Progression-free survival **(B)** in the S-TACE and complications of transarterial chemoembolization (TACE) groups in patients with early diffuse recurrence (EDR). Kaplan-Meier curves showed overall survival **(C)** and Progression-free survival **(D)** in the S-TACE and complications of transarterial chemoembolization (TACE) groups for patients with late diffuse recurrence (LDR).

In the EDR group, median OS was 17.5 months (95% CI: 14.3, 19.7 months) and 11.0 months (95% CI: 9.9, 12.1 months) in the S-TACE and TACE monotherapy groups, respectively. The median PFS was 5.0 months (95%CI: 3.9, 6.1 months) and 4.0 months (95% CI: 3.4, 4.6 months) in the S-TACE and TACE monotherapy groups, respectively. There was a significant difference in OS and PFS (log-rank test, *p*=0.011 and *p*=0.049, respectively) between the two groups (**Figure 3**).

In LDR group, median OS was 24.0 months (95% CI: 19.1, 28.9 months) and 16.0 months (95% CI: 14.6, 17.4 months) in S-TACE and TACE monotherapy groups, respectively. The median PFS was 8.0 months (95% CI: 6.9, 9.1 months) and 5.0 months (95% CI: 4.3, 5.7 months) in the S-TACE and TACE monotherapy groups, respectively. There was a significant difference in OS (log-rank test, *p*=0.044) between the two groups, but not for PFS (log-rank test, *p*= 0.176) (**Figure 3**).

**Figure 4:**
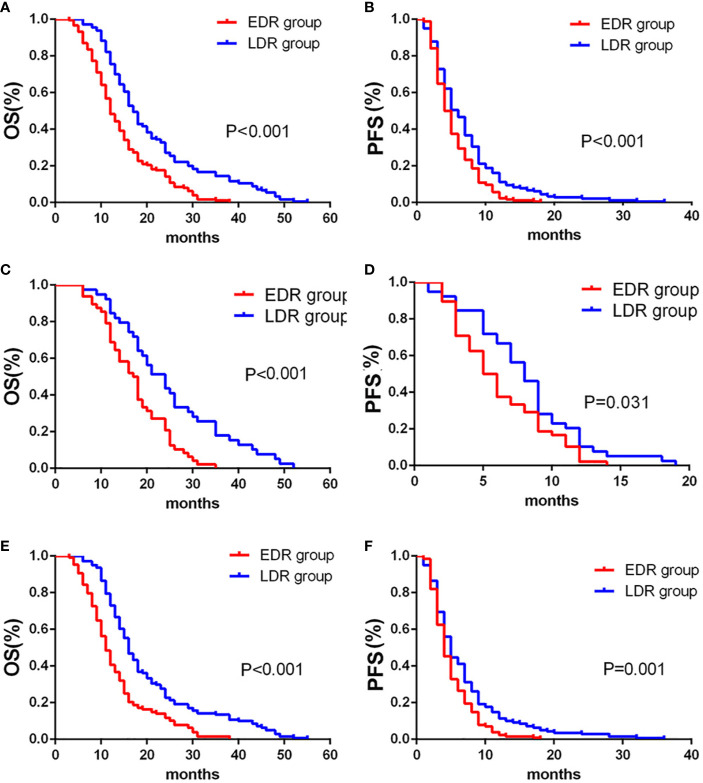
Kaplan-Meier curves showed overall survival **(A)** and Progression-free survival **(B)** in the early diffuse recurrence (EDR) and late diffuse recurrence (LDR) groups. Kaplan-Meier curves showed overall survival **(C)** and Progression-free survival **(D)** in the EDR and LDR groups for patients with S-TACE. Kaplan-Meier curves showed overall survival **(E)** and Progression-free survival **(F)** in the EDR and LDR groups for patients with complications of transarterial chemoembolization (TACE) alone.

Overall, the LDR group had better OS and PFS (p< 0.001) than the EDR group. In subgroup analysis, the LDR group had better OS (*p*<0.001) and PFS (*p*=0.031) than the EDR group when S-TACE was performed. When TACE was performed, the LDR group had better OS (*p*<0.001) and PFS (*p*<0.001) than the EDR group (**Figure 4**).

### Univariate and Multivariate Analysis

Univariate analysis of EDR patients found that the factors related to OS were HBV-DNA and maximum tumor size (*p* = 0.023 and 0.008, respectively) (**Table 3**). Multivariate analysis found that HBV-DNA and maximum tumor size were found to be independent predictors of poor OS in EDR patients (*p*= and 0.030 and 0.010, respectively) (**Table 3**).

**Table 3:** Univariable and multivariable analysis of prognostic factors for OS in early diffuse recurrence (EDR) patients.

Factor	Univariate analysis			Multivariate analysis		
	P Value	Hazard ratio	95%CI	P Value	Hazard ratio	95%CI
**Sex**	0.912	0.972	(0.583, 1.620)	NA	—	—
**Age**	0.991	0.998	(0.730, 1.364)	NA	—	—
**Cause of HCC**	0.949	1.007	(0.822, 1.233)	NA	—	—
**Liver cirrhosis**	0.752	0.946	(0.668, 1.338)	NA	—	—
**AFP**	0.143	1.257	(0.926, 1.707)	NA	—	—
**ALB**	0.380	0.989	(0.964, 1.014)	NA	—	—
**TB**	0.460	1.004	(0.993, 1.016)	NA	—	—
**HBV-DNA**	0.023	1.424	(1.049, 1.933)	0.030	1.403	(1.032,1.906)
**ECOG**	0.196	1.338	(0.861, 2.080)	NA	—	—
**Child-Pugh**	0.462	0.823	(0.490, 1.383)	NA	—	—
**Maximum tumor size**	0.008	1.500	(1.112, 2.025)	0.010	1.482	(1.097,2.002)

–, no data; CI, confidence interval; NA, not applicable.

Univariate analysis found that in the LDR group, AFP, TB, HBV-DNA, and maximum tumor size were associated with OS (*p*=0.002, 0.041, 0.038, and 0.003, respectively) (**Table 4**). In multivariate regression analysis, AFP, TB, and maximum tumor size were found to be independent predictors of poor OS in LDR patients (p=0.008, 0.043, 0.045) (**Table 4**).

**Table 4:** Univariable and multivariable analysis of prognostic factors for OS in late diffuse recurrence (LDR) patients.

Factor	Univariate analysis			Multivariate analysis		
	P Value	Hazard ratio	95%CI	P Value	Hazard ratio	95%CI
**Sex**	0.998	1.001	(0.605, 1.654)	NA	—	—
**Age**	0.242	0.992	(0.979, 1.005)	NA	—	—
**Cause of HCC**	0.709	0.917	(0.581, 1.447)	NA	—	—
**Liver cirrhosis**	0.719	0.937	(0.656, 1.338)	NA	—	—
**AFP**	0.002	1.621	(1.189, 2.208)	0.008	1.545	(1.122, 2.126)
**ALB**	0.066	1.330	(0.981, 1.803)	NA	—	—
**TB**	0.041	0.698	(0.495, 0.985)	0.043	0.698	(0.494, 0.988)
**HBV-DNA**	0.038	1.384	(1.018, 1.879)	0.091	1.310	(0.957, 1.793)
**ECOG**	0.627	0.900	(0.589, 1.377)	NA	—	—
**Child-Pugh**	0.535	0.870	(0.561, 1.350)	NA	—	—
**Maximum tumor size**	0.003	1.584	(1.166, 2.154)	0.045	1.389	(1.007, 1.915)

–, no data; CI, confidence interval; NA, not applicable.

### Subgroup Analysis

**Figure 5:**
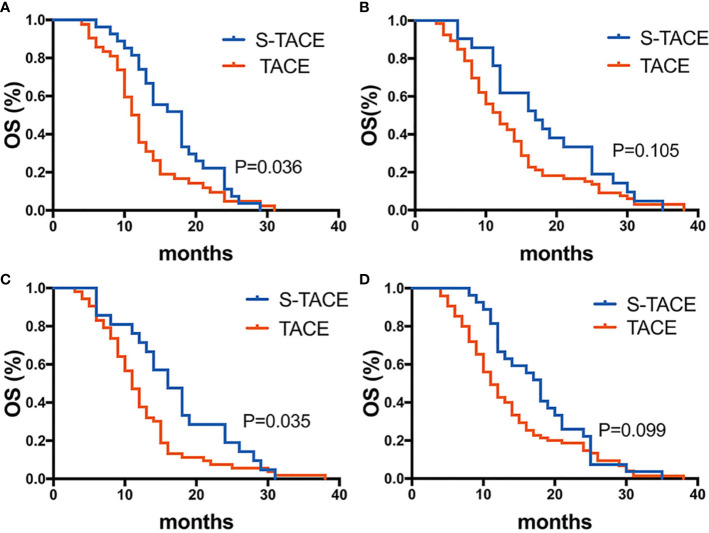
Kaplan-Meier curves showed overall survival in the S-TACE and complications of transarterial chemoembolization (TACE) groups in patients with early diffuse recurrence (EDR) [**(A)** maximum diameter of tumor size > 5cm, **(B)** maximum diameter of tumor size < 5cm, **(C)** HBV-DNA>100, **(D)** HBV-DNA<100].

**Figure 6:**
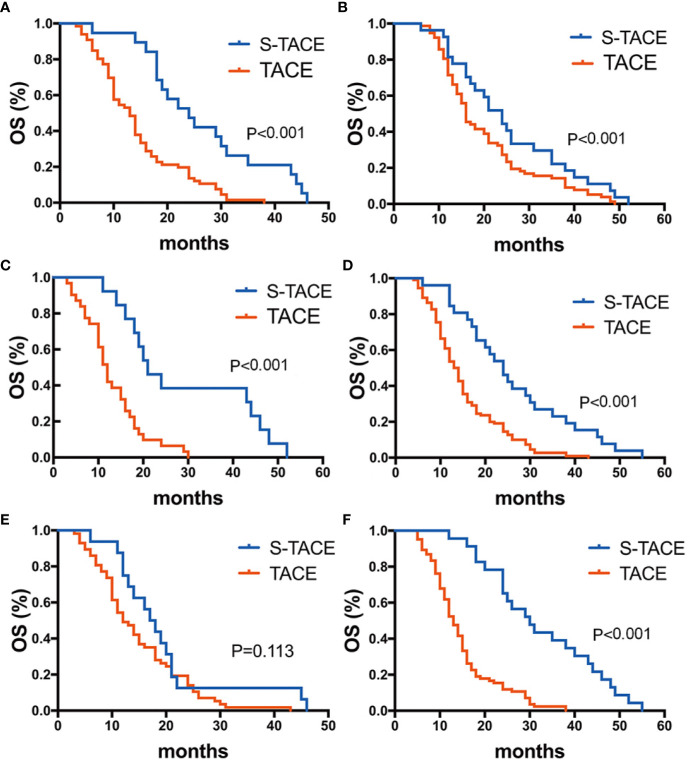
Kaplan-Meier curves showed overall survival in the S-TACE and complications of transarterial chemoembolization (TACE) groups in patients with late diffuse recurrence (LDR) [**(A)** AFP≥400 ng/ml, **(B)** AFP<400 ng/ml, **(C)** TB>28 g/L, **(D)** TB < 28 g/L, **(E)** maximum diameter of tumor size> 5 cm, **(F)** maximum diameter of tumor size< 5 cm].

In EDR patients, S-TACE therapy resulted in higher OS than TACE monotherapy in tumors with a maximum diameter of >5 cm and HBV-DNA>100 group *(p*= 0.036 and 0.035, respectively). There was no significant difference between S-TACE and TACE monotherapy in tumors with a maximum diameter of < 5 cm and HBV<100 group (*p*=0.105 and 0.099, respectively) (**Figure 5**). In LDR patients, patients given S-TACE therapy had better OS than those given TACE monotherapy in patients with AFP≥400 ng/ml, AFP<400 ng/ml, TB<28 g/L, TB>28 g/L, and maximum diameter of tumor < 5 cm group (p<0.001,<0.001, <0.001, <0.001, and <0.001, respectively). There was no significant difference between patients given S-TACE and TACE monotherapy with tumors > 5 cm (p=0.113) (**Figure 6**).

### Complications

There were no deaths in either the EDR or LDR group within 30 days after treatment. Major and minor complications are reported in **Tables 5** and **6**. There was no significant difference in major complications between the EDR and LDR groups (33.5% vs. 28.2%, *p* = 0.69). By the end of the follow-up period, 47 patients (53%) had discontinued sorafenib administration, including 24 (50%) patients in the EDR group and 23 (59%) patients in the LDR group because of serious AEs. Most patients (97.9%) experienced at least one AE (see **Table 5** and **Table 6**).

**Table 5:** Number (percentage) of patients reporting AEs in early diffuse recurrence (EDR) patients by CTCAE grading^1^.

Adverse Event	S-TACE (n=48)		TACE Monotherapy (n=128)		P Value
	Grade 1–2	Grade 3–4	Grade 1–2	Grade 3–4	
**Diarrhea**	23(47.9)	4(8.3)	67(52.3)	10(7.8)	0.605
**Abdominal pain**	29(60.4)	7(14.6)	77 (60.2)	13 (10.2)	0.810
**Ascites**	5(10.4)	1(2.1)	13(10.2)	3(2.4)	0.995
**Vomiting**	30(62.5)	1(2.1)	93 (72.7)	4(3.2)	0.169
**Fatigue**	34(70.8)	2(4.2)	77(60.2)	4(3.2)	0.167
**Fever**	34(70.8)	3(6.3)	102 (79.7)	8 (6.3)	0.189
**Headache**	7(14.6)	1(2.1)	13 (10.2)	3 (2.4)	0.461
**Upper respiratory infection**	5 (10.5)	0(0)	10 (7.8)	1(0.8)	0.696
**PLT decreased**	9(18.8)	2(4.2)	16(12.5)	6(4.5)	0.360
**WBC decreased**	8(16.7)	3 (6.3)	19(14.8)	4(3.2)	0.500
**Anaemia**	4 (8.3)	1(2.1)	13(10.2)	3 (2.4)	0.703
**Hand-foot skin reaction**	31 (64.6)	16 (33.3)	—	—	NA
**Hypertension**	13(27.1)	4 (8.3)	—	—	NA
**Hair loss**	7 (14.6)	0 (0)	—	—	NA
**Gastrointestinal hemorrhage**	6(12.6)	3(6.3)	—	—	NA
**Epistaxis**	7 (14.6)	0 (0)	—	—	NA

CTCAE grading^1^: National Cancer Institute (NCI) Common Terminology Criteria for Adverse Events (CTCAE) version 4.0; –, no data; NA, not applicable; PLT, Platelets; WBC, White blood cell.

**Table 6:** Number (percentage) of patients reporting AEs in late diffuse recurrence (LDR) patients by CTCAE grading^1^.

Adverse Event	S-TACE (n=39)		TACE Monotherapy (n=141)		P Value
	Grade 1–2	Grade 3–4	Grade 1–2	Grade 3–4	
**Diarrhea**	21(53.8)	2(5.2)	63(44.7)	9(6.4)	0.332
**Abdominal pain**	25(64.1)	6(15.4)	95(67.4)	28(19.9)	0.537
**Ascites**	4(10.2)	2(5.1)	18(12.8)	5(3.5)	0.855
**Vomiting**	11(28.2)	3(7.7)	31(21.9)	6(4.3)	0.266
**Fatigue**	19(48.7)	3(7.7)	69(48.9)	10(7.1)	0.994
**Fever**	16(41.0)	4(10.4)	59(41.8)	9(6.4)	0.882
**Headache**	7(17.9)	0(0)	21(14.9)	3(2.1)	0.849
**Upper respiratory infection**	4(10.4)	0(0)	8(5.7)	2(1.4)	0.496
**PLT decreased**	6(15.4)	1(2.6)	18(12.8)	6(4.3)	0.855
**WBC decreased**	5(12.8)	0(0)	13(9.2)	3(2.1)	0.765
**Anaemia**	3(7.7)	0(0)	16(11.3)	5(3.5)	0.262
**Hand-foot skin reaction**	28(71.8)	9(23.1)	—	—	NA
**Hypertension**	11(28.2)	4(10.4)	—	—	NA
**Hair loss**	3(7.7)	0(0)	—	—	NA
**Gastrointestinal hemorrhage**	4(10.4)	2(5.1)	—	—	NA
**Epistaxis**	3(7.7)	0(0)	—	—	NA

CTCAE grading^1^: National Cancer Institute (NCI) Common Terminology Criteria for Adverse Events (CTCAE) version 4.0; –, no data; NA, not applicable; PLT, Platelets; WBC, White blood cell.

In the EDR group, the major AEs that were experienced by at least 10% of patients were abdominal pain (11.3%) and hand-foot skin reaction (33.3%). Others major AEs included diarrhea (8.0%), hypertension (8.3%), gastrointestinal hemorrhage (6.3%), and fever (6.3%). There was no significant difference in occurrence of AEs between the S-TACE and TACE monotherapy groups (**Table 5**). In the LDR group, the major AEs that were experienced by at least 10% of patients were abdominal pain (18.9%), hand-foot skin reaction (23.1%), and hypertension (10.4%). Others major AEs included vomiting (5.0%), fever (7.2%), fatigue (7.2%), diarrhea (6.1%), ascites (3.9%), and gastrointestinal hemorrhage (5.1%). There was no significant difference in occurrence of AEs between the S-TACE and TACE monotherapy groups (**Table 6**). Most abdominal pain was caused by the TACE therapy, and could be relieved by morphine or flurbiprofen axetil. Hand-foot skin reactions and hypertension were caused by sorafenib. About half of the hand-foot skin reactions were relieved by using lubricant or regressed within several months. Angiotensin receptor blockers were used to effectively relieve hypertension caused by sorafenib. All major AEs were treated without AE-related death and patients recovered with in two weeks.

## Discussion

Recurrence rate after liver resection is high in HCC patients (5–7, 18). A short interval from resection of HCC to recurrence leads to worse outcomes. Most patients with recurrence were not eligible for repeated hepatectomy due to being DR patients. TACE is the optimal treatment method for these patients (18, 19). In addition, sorafenib is recommended for unresectable HCC. Recently, various reports have shown that the combination of TACE and sorafenib resulted in better clinical outcomes than TACE monotherapy in multinodular HCC without vascular invasion or extra hepatic spread (9, 10, 13). Our study revealed that compared with TACE monotherapy, S-TACE could effectively prolong OS in EDR and LDR patients, which was consistent with the results of abovementioned previous studies.

Our data indicated that S-TACE could significantly improve OS in patients with EDR and LDR. Jung Ho Park reported that postoperative early multinodular recurrence was associated with the presence of portal vein tumor thrombi and intrahepatic metastases, and this form of recurrence was found to have a grave prognosis compared with that in late multinodular recurrence (20). Our findings supported these results and suggested that multiple comprehensive treatments should be applied in these patients. In addition, TACE induced ischemic or hypoxic changes which led to increased VEGF activity in surviving cancer tissue (21). Therefore, the use of a potent multikinase inhibitor, such as sorafenib, could limit the proliferative, proangiogenic, and/or antiapoptopic effects of VEGF expression, which could restrict tumor growth after TACE (12). This result corroborated the findings of a previous study reporting that S-TACE was suitable for metachronous, multicentric HCC nodules (22). Our study also demonstrated that S-TACE could improve the efficacy of multinodular recurrence.

In our study, the S-TACE combination showed little advantage over TACE monotherapy in LDR patients. This may be related to the fact that late recurrence is usually associated with underlying liver conditions, such as cirrhosis or active hepatitis (23). Patients with late recurrence might die due to poor liver functions; thus, the advantages of S-TACE would not be present in these patients. Thus, anti-viral therapy and liver protection should be recommended for these patients. In addition, multiple tumors, satellite nodules, and tumors greater than 5 cm were independent risk factors for late recurrence according to Xu et al. (24). Thus, S-TACE should be employed early for patients when the tumor load is high.

In terms of risk factors for EDR and LDR, we found that tumor size and HBV-DNA were associated with EDR, while AFP, TB, and tumor size were related to LDR. This could be related to the larger maximum tumor size and higher AFP level, which indicated a higher tumor load leading to poor prognosis (25, 26). Therefore, combined therapies should be applied to higher tumor loads, which would help to improve OS. Additionally, our study indicated that the OS of LDR patients was much longer than that of EDR patients, regardless of the treatment. This may be due to EDR recurrence being accompanied by more malignant biological behaviors.

In our study, most AEs in the LDR or EDR group through S-TACE or TACE monotherapy were grade 1 or 2 and could be easily controlled. These results were consistent with those of the previous studies (13, 27, 28). Importantly, no lethal AEs were reported in patients with S-TACE, and all major serious AEs were in remission after treatment. Our observations were consistent with those of previous studies reporting that S-TACE was not associated with a significantly greater incidence/severity of adverse events than TACE monotherapy (29). Most patients in both the LDR and EDR groups discontinued sorafenib because of serious AEs. Serious hand-foot skin reaction and abdominal pain were common reasons for discontinuation of sorafenib. Since the mechanism of hand-skin reaction and abdominal pain was unknown, they were difficult to treat.

Our study had several limitations. First, this study was retrospective, which may reduce the reliability of data leading to selection bias. Second, EDR and LDR were not determined histologically or genetically. So, it was difficult to explain why there were different prognoses and risk factors in LDR and EDR patients, and we could not provide individualized treatment. We suggest that genome sequencing of HCC would be a key next step for this research. Third, the limited number of patients were insufficient for subgroup analysis. Consequently, a multi-center prospective randomized controlled trial is needed to confirm our findings. Fourth, the administration of sorafenib could be improved in this study; we should closely follow-up with patients and take positive measures when serious AEs occur. Finally, a higher rate of patients discontinuing sorafenib could influence the effect of S-TACE.

In conclusion, S-TACE resulted in improved outcomes in EDR patients including OS and PFS, especially in patients with a maximum tumor diameter > 5 cm and HBV-DNA>100, in comparison with TACE monotherapy. In LDR patients, there was significantly better OS in the S-TACE group, especially for patients with AFP>400 mg/L, AFP<400 mg/L, TB>28 g/L, TB<28 g/L, and a maximum tumor diameter > 5 cm.

## Data Availability Statement

The original contributions presented in the study are included in the article/supplementary materials, further inquiries can be directed to the corresponding authors.

## Ethics Statement

The studies involving human participants were reviewed and approved by the First Affiliated Hospital of Sun Yat-Sen University. The patients/participants provided their written informed consent to participate in this study.

## Author Contributions

WY, JL, and WF conceptualized the study and contributed to the investigation. WY, MX, and ML performed the formal analysis, were in charge of the projection administration, and wrote the original draft. YQW and YZ were in charge of the data curation and methodology. YW was in charge of the software. All authors contributed to the article and approved the submitted version.

## Conflict of Interest

The authors declare that the research was conducted in the absence of any commercial or financial relationships that could be construed as a potential conflict of interest.
